# Protective Effects from Prior Pneumococcal Vaccination in Patients with Chronic Airway Diseases during Hospitalization for Influenza—A Territory-Wide Study

**DOI:** 10.3390/vaccines12070704

**Published:** 2024-06-23

**Authors:** Wang-Chun Kwok, David Christopher Lung, Terence Chi-Chun Tam, Desmond Yat-Hin Yap, Ting-Fung Ma, Chung-Ki Tsui, Ru Zhang, David Chi-Leung Lam, Mary Sau-Man Ip, James Chung-Man Ho

**Affiliations:** 1 Department of Medicine, The University of Hong Kong, Queen Mary Hospital, 102 Pokfulam Road, Pokfulam, Hong Kong SAR, China; kwokwch@hku.hk (W.-C.K.); tcctam@netvigator.com (T.C.-C.T.); desmondy@hku.hk (D.Y.-H.Y.); anna316@hku.hk (C.-K.T.); u3590587@connect.hku.hk (R.Z.); dcllam@hku.hk (D.C.-L.L.); msmip@hku.hk (M.S.-M.I.); 2 Department of Pathology, Queen Elizabeth Hospital, 30 Gascoigne Road, Kowloon, Hong Kong SAR, China; lungdc@ha.org.hk; 3 Department of Statistics, University of South Carolina, Columbia, SC 29208, USA; tingfung@mailbox.sc.edu

**Keywords:** influenza, asthma, bronchiectasis, COPD, pneumococcal vaccine

## Abstract

Influenza is an important respiratory viral pathogen in adults, with secondary bacterial pneumonia being a common complication. While pneumococcal vaccines can prevent pneumococcal pneumonia and invasive pneumococcal disease, whether they can also prevent the severe in-hospital outcomes among patients hospitalized for influenza has not been examined. A territory-wide retrospective study was conducted in Hong Kong, which included all adult patients having chronic airway diseases (asthma, bronchiectasis, and chronic obstructive pulmonary disease) hospitalized for influenza and who had received seasonal influenza vaccine. The occurrence of secondary bacterial pneumonia, mortality, and other severe in-hospital outcomes were compared among subjects with or without pneumococcal vaccination. There was a total of 3066 eligible patients who were hospitalized for influenza in public hospitals in Hong Kong from 1 January 2016 to 30 June 2023. Completed pneumococcal vaccination with PSV23/PCV13 conferred protection against secondary bacterial pneumonia, all-cause mortality, and respiratory cause of mortality with adjusted odds ratios of 0.74 (95% CI = 0.57–0.95, *p* = 0.019), 0.12 (95% CI = 0.03–0.53, *p* = 0.005), and 0.04 (95% CI = 0.00–0.527, *p* = 0.0038), respectively.

## 1. Introduction

Influenza is one of the most important viruses causing acute respiratory illness with seasonal outbreaks [1,2]. Pneumonia is a common complication of influenza, which can be due to primary influenza pneumonia, secondary bacterial pneumonia, and mixed viral and bacterial pneumonia [3]. Secondary bacterial pneumonia was differentiated from mixed viral and bacterial pneumonia by the chronological sequence of the infections, whether it was a preceding or concurrent viral respiratory tract infection [4]. The incidence of secondary bacterial pneumonia is reported to be five times higher among older individuals and those with underlying co-morbidities [5], with the onset within the first six days of influenza infection [6]. The common bacterial pathogens causing secondary bacterial pneumonia include *Streptococcus pneumoniae* (*S. pneumoniae*), *Staphylococcus aureus*, and group A *Streptococcus* [7]. In patients with chronic obstructive pulmonary disease (COPD), Gram-negative bacteria such as *Enterobacteriaceae* and *Pseudomonas aeruginosa* (*P. aeruginosa*) are also frequently isolated in COPD exacerbation and pneumonia [8]. Respiratory viral–bacterial co-infection has been reported to be associated with increased mortality and morbidity as compared to a viral or bacterial infection alone in elderly and chronically ill patients [4,9].

Patients with chronic respiratory diseases, including but not limited to asthma, bronchiectasis, and COPD, are at risk of severe influenza and are recommended to receive influenza as well as pneumococcal vaccines [10,11,12].

Influenza and pneumococcal vaccines on their own have been proven to be effective among patients with chronic respiratory diseases [13,14,15,16,17,18,19]. And it has also been reported that the additive inoculation of the influenza vaccine and 23-valent pneumococcal vaccine (PSV23) in Japanese patients with chronic respiratory diseases could prevent the development of bacterial respiratory infections [20]. Influenza and PSV23 vaccination, separately or together, can also reduce the risk of COPD exacerbation, pneumonia, and related hospitalization [21].

While it is not unexpected that receiving both influenza and pneumococcal vaccines can prevent pneumonia in the long run, whether receiving both of them, in particular, with different pneumococcal vaccines, can prevent adverse outcomes upon hospitalization for influenza has not been reported. In light of this, we conducted this retrospective territory-wide study to assess the benefits of pneumococcal vaccines, namely, the PSV23 and 13-valent pneumococcal conjugate vaccine (PCV13) that are covered in the Government Vaccination Programme in Hong Kong, in patients with chronic airway diseases (asthma, bronchiectasis, and COPD), who had already received the seasonal influenza vaccine.

## 2. Materials and Methods

Adult patients with chronic airway diseases who had already received seasonal influenza vaccine and were admitted to public hospitals in Hong Kong for influenza from 1 January 2016 to 30 June 2023 were included. The eligible patients were identified from Clinical Data Analysis and Reporting System (CDARS) of Hospital Authority using International Classification of Diseases (Ninth Revision) code of 487.8 for influenza. The CDARS is an electronic health record (EHR) database managed by the Hong Kong Hospital Authority (HKHA), which is a public healthcare service provider with 43 hospitals and institutions, and 122 outpatient clinics, covering more than 90% of the Hong Kong population since 1993 [22,23,24]. The CDARS captures medical information including diagnosis, drug prescription details, demographics, admissions, medical procedures, and laboratory results. The study was approved by the Institutional Review Board (IRB) of the University of Hong Kong and Hospital Authority Hong Kong West Cluster (UW 24-137) and all methods were performed in accordance with the relevant guidelines and regulations provided by the IRB. Patient informed consent was waived in this retrospective study by the IRB as there was no active patient recruitment and the data were already de-identified. The study was conducted in compliance with the Declaration of Helsinki.

Inclusion criteria were age of 18 years old or above, with underlying asthma, bronchiectasis, or COPD, having previously received seasonal influenza vaccine, and being hospitalized for laboratory-confirmed influenza. Patients with viral co-infection were excluded.

Secondary bacterial pneumonia was defined as the compatible radiological changes on chest radiograph with supporting laboratory parameters (leukocytosis and neutrophilia) that necessitate systemic antibiotic treatment. Secondary pneumococcal pneumonia was defined as fulfilling the criteria of secondary bacterial pneumonia, and with the microbiological evidence, which included the isolation of *S*. *pneumoniae* from sputum or other respiratory tract specimens; or having a positive urine pneumococcal antigen. IPD was defined as infections in which *S*. *pneumoniae* is isolated from a normally sterile body site, which included bacteremia, meningitis, spontaneous bacterial peritonitis, septic arthritis, endocarditis, spinal epidural abscess, osteomyelitis, and dental abscess.

### 2.1. Procedures

The first main exposure of interest was the pneumococcal vaccination status, including no pneumococcal vaccine, PSV23 alone, PCV13 alone, and both PSV23 and PCV13. The primary outcome was the development of secondary bacterial pneumonia. The secondary outcomes included (1) all-cause mortality within the index hospitalization; (2) mortality from respiratory cause within the index hospitalization; (3) development of pneumococcal pneumonia; (4) development of invasive pneumococcal disease (IPD); (5) development of severe respiratory failure requiring invasive (IMV) or non-invasive mechanical ventilation (NIV) (SRF); and (6) intensive care unit (ICU) admission. Regular use of inhaled corticosteroid (ICS), long-acting beta-agonists (LABAs), and long-acting muscarinic antagonists (LAMAs) were defined as continuous use for at least 12 months within the study period. Non-pneumococcal/other secondary bacterial pneumonia was defined as secondary bacterial pneumonia from all other bacteria except *S*. *pneumoniae* or from undetermined micro-organisms, which were identified from sputum or other respiratory tract specimens.

### 2.2. Statistical Analysis

Descriptive tables were created to compare incidence of the outcomes stratified by pneumococcal vaccination status, with demographic and clinical data described in actual frequency or mean ± standard deviation (SD), or median [inter-quartile range (IQR)] when appropriate. Baseline demographic and clinical data were compared between the patients in different subgroups by one-way ANOVA for continuous variables and chi-square test for categorical variables. Multiple imputation by chained equations were used to impute missing data for variables included in the adjusted analysis model. To identify whether pneumococcal vaccines were associated with the development of primary and secondary outcomes, univariate logistic regression analyses were performed. The group who did not receive PSV23 and PCV13 were chosen as the control group for comparison. Next, we assessed the adjusted association between pneumococcal vaccines with the development of primary and secondary outcomes by controlling for confounding variables, which included age, sex, ethnicity, baseline CCI, types of chronic airway diseases, presence of diabetes mellitus (DM), cardio-/cerebro-vascular diseases, history of malignancies, and the use of ICS, LABAs, and LAMAs. Firth logistic regression was used in case the event rate was 0 in the subgroup.

Sensitivity analyses were conducted (1) among patients aged 65 or above, who were eligible to receive free influenza and pneumococcal vaccines by government policy, regardless of the presence of co-morbidities, and (2) comparing patients who did not receive influenza/PSV23/PCV13 and the other three subgroups that were included in the primary analysis.

Subgroup analyses were performed (1) among patients who received the wrong season’s vaccine and those who received a vaccine matched to the influenza strain, and (2) patients who received PCV13 first or PPV23 first, if they received both PCV13/PSV23.

Data analyses were performed using 28th version of IBM^®^ SPSS^®^ statistical package. For all statistical analyses, statistical significance was assessed at an α level of 0.05. STROBE and RECORD reporting guidelines were followed in the generation of this report.

## 3. Results

### 3.1. Patients’ Characteristics

There were 41,206 adult patients hospitalized for influenza infection in public hospitals in Hong Kong from 1 January 2016 to 30 June 2023. Among them, 3066 had pre-existing chronic airway diseases and received an annual seasonal influenza vaccine in the past 12 months before the index admission; 3116 patients with pre-existing chronic airway diseases did not receive an annual seasonal influenza vaccine in the past 12 months before the index admission, in which 3027 of them also did not receive any pneumococcal vaccines. In the 3066 patients with pre-existing chronic airway diseases and received an annual seasonal influenza vaccine in the past 12 months before the index admission, about half of these patients (n = 1521; 49.6%) did not have a pneumococcal vaccine, 133 (4.3%) had PSV23 only, 1091 (35.6%) had PCV13 only, and 321 (10.5%) had both the PSV23 and PCV13 vaccination. In the ‘PSV23 only’ group, eight of them received two doses and the remaining received only one dose of PSV23. In the ‘both PSV23/PCV13’ group, five of them received two doses and the remaining received only one dose of PSV23. In the ‘both PSV23/PCV13’ group, 227 (70.7%) of them received PCV13 first with the mean interval between PCV13 and PSV23 being 16.8 ± 5.7 months; 94 (29.3%) of them received PSV23 first with the mean interval between PCV13 and PSV23 being 26.7 ± 8.4 months.

**Figure 1:**
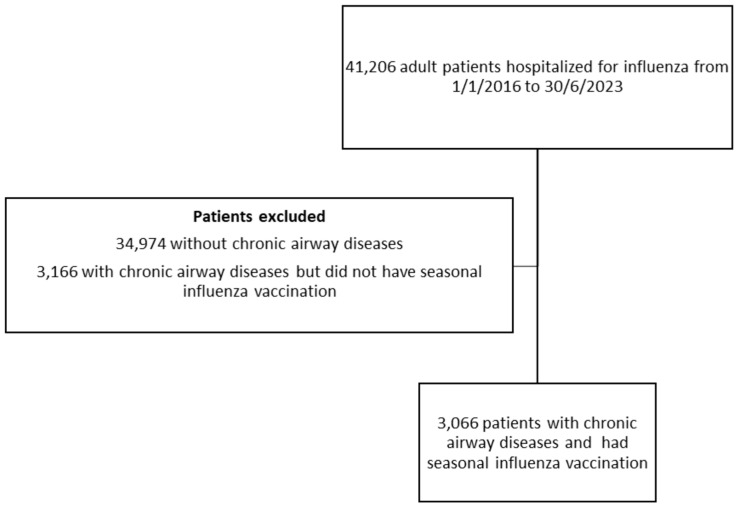
The patient selection flow chart.

The baseline demographics of these patients are listed in Table 1. The patient selection is illustrated in Figure 1.

The number of patients who had pre-existing chronic airway diseases and received an annual seasonal influenza vaccine in the past 12 months before the index admission that were hospitalized for influenza had a drastic reduction from the year 2020 to 2022, which increased again in the year 2023, likely reflecting the impact of coronavirus disease 2019 (COVID-19) [25,26]. The admission number in each year was illustrated in Supplementary Figure S1.

### 3.2. Primary Outcome—Secondary Bacterial Pneumonia

**Figure 2:**
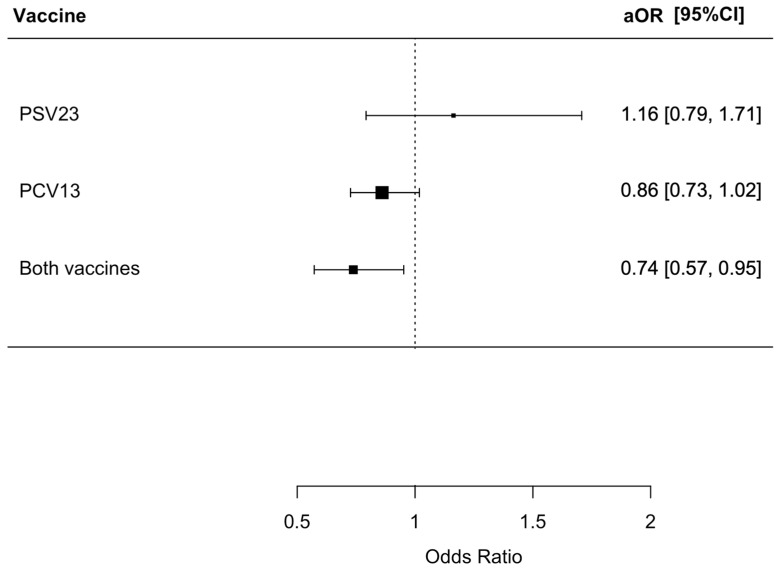
Risk of secondary bacterial pneumonia in patients receiving different pneumococcal vaccines. The group who did not receive PSV23 and PCV13 was chosen as the control group for comparison.

There were 1818 (59.3%) patients in this cohort who developed secondary bacterial pneumonia in the index admission for influenza; 411 of them had a urine pneumococcal antigen test performed with 29 positive results. Among these 29 patients with a positive urine pneumococcal antigen test result, 27 also had *S. pneumoniae* isolated in the sputum, while two of them only had a positive urine pneumococcal antigen test result but a negative sputum culture. Among the patients, 702 of them had a urine legionella antigen test performed with two positive results; 313 patients had procalcitonin level measured. The median procalcitonin level was 0.12 (0.05–0.59) ng/mL in the group with secondary bacterial pneumonia and 0.08 (0.05–0.27) ng/mL in the group without secondary bacterial pneumonia. Among those with no pneumococcal vaccination, PSV23 only, PCV13 only, and both PSV23 and PCV13, there were 928 (61.0%), 86 (64.7%), 640 (58.7%), and 164 (51.1%) patients who developed secondary bacterial pneumonia (*p* = 0.006). Patients who received both PSV23 and PCV13 had a statistically lower risk of developing secondary bacterial pneumonia with an odds ratio (OR) of 0.67 (95% confidence interval [CI] = 0.52–0.85, *p* = 0.001), while those who received only PCV13 or PSV23 did not have a risk reduction in developing secondary bacterial pneumonia with an OR of 0.91 (95% CI = 0.77–1.06, *p* = 0.23) and an OR of 1.17 (95% CI = 0.81–1.69, *p* = 0.41), respectively. The adjusted OR (aOR) for patients who received both PSV23 and PCV13 were 0.74 (95% CI = 0.57–0.95, *p* = 0.019). The results are summarized in Figure 2. The microbiology of the micro-organisms identified is summarized in Supplementary Table S1.

### 3.3. Secondary Outcomes—Mortality, Pneumococcal Disease, Severe Respiratory Failure, and ICU Care

**Figure 3:**
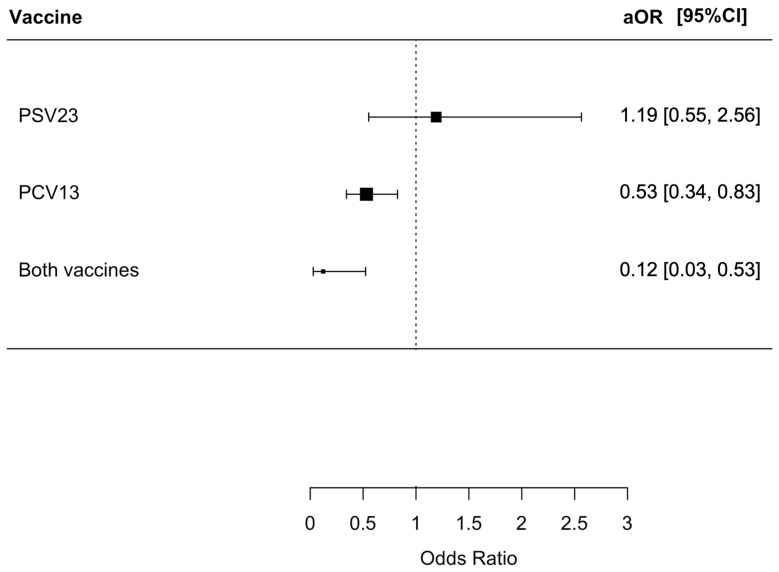
The risk of all-cause mortality in index admission in the patients receiving different pneumococcal vaccine.: The group who did not receive PSV23 and PCV13 was chosen as the control group for comparison.

We found that 127 (4.1%) patients died from various causes within the index admission, with 87 (5.7%), 8 (6.0%), 30 (2.7%), and 2 (0.6%) having no PSV23/PCV13, only PSV23, only PCV13, and both PSV23/PCV13 vaccinations, respectively (*p* < 0.001). Patients who received both PSV23/PCV13 and PCV13 only had a statistically lower risk of all-cause mortality with an OR of 0.10 (95% CI = 0.03–0.42, *p* = 0.002) and 0.47 (95% CI = 0.31–0.71, *p* < 0.001), respectively. The aOR for patients who received both PSV23/PCV13 and PCV13 were only 0.12 (95% CI = 0.03–0.53, *p* = 0.005) and 0.53 (95% CI = 0.34–0.83, *p* = 0.005), respectively. The results are summarized in Figure 3.

**Figure 4:**
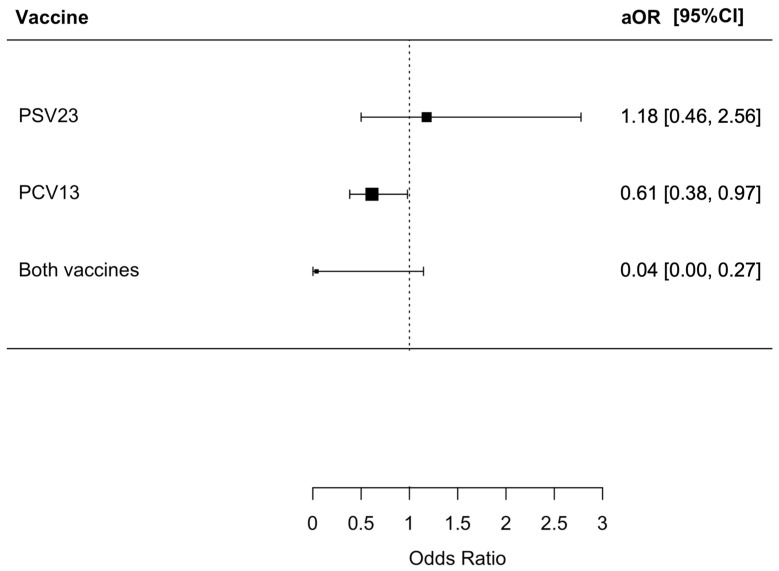
Risk of respiratory mortality in index admission in patients receiving different pneumococcal vaccines.: The group who did not receive PSV23 and PCV13 was chosen as the control group for comparison.

We found that 100 (4.1%) patients died from respiratory causes during the index admission, with 67 (4.4%), 6 (4.5%), 27 (2.5%), and 0 (0%) having no PSV23/PCV13, only PSV23, only PCV13, and both PSV23/PCV13 vaccinations, respectively (*p* < 0.001). Patients who received both PSV23/PCV13 and PCV13 only had a statistically lower risk of respiratory mortality with an OR of 0.034 (95% CI = 0.00–0.23, *p* = 0.002) and 0.56 (95% CI = 0.35–0.86, *p* = 0.008), respectively. The aOR for patients who received both PSV23/PCV13 and PCV13 were only 0.04 (95% CI = 0.00–0.27, *p* = 0.0038) and 0.61 (95% CI = 0.38–0.97, *p* = 0.036), respectively. The results are summarized in Figure 4.

We found that 148 (4.8%) developed confirmed secondary pneumococcal pneumonia, with 66 (4.3%), 6 (4.5%), 58 (5.3%), and 18 (5.6%) having no PSV23/PCV13, only PSV23, only PCV13, and both PSV23/PCV13 vaccinations, respectively (*p* = 0.61). There was no statistically significant difference in the risk of developing secondary pneumococcal pneumonia across all four subgroups.

We found that 4 (4.8%) developed IPD, with 3 (0.2%), 0 (0%), 1 (0.1%), and 0 (0%) having no PSV23/PCV13, only PSV23, only PCV13, and both PSV23/PCV13 vaccinations, respectively (*p* = 0.74). There is no statistically significant difference for the risks of developing IPD across the four subgroups, but the overall IPD incidence rate is low in this cohort.

We found that 1024 (33.4%) developed SRF, with 509 (33.5%), 47 (35.3%), 359 (32.9%), and 109 (34.0%) having no PSV23/PCV13, only PSV23, only PCV13, and both PSV23/PCV13 vaccinations, respectively (*p* = 0.94). There was no statistically significant difference in the risk of developing SRF across all four subgroups.

We found that 19 (0.6%) required ICU admission, with 10 (0.7%), 1 (0.8%), 7 (0.6%), and 1 (0.3%) having no PSV23/PCV13, only PSV23, only PCV13, and both PSV23/PCV13 vaccinations, respectively (*p* = 0.90). There was no statistically significant difference in the risk of ICU admission across all four subgroups.

### 3.4. Sensitivity Analysis

A sensitivity analysis was performed for patients from the older age group (≥65 years old), who are currently eligible for free pneumococcal vaccines by the Hong Kong Government Vaccination Programme. There was a total of 2664 patients aged 65 or above hospitalized for influenza. Again, patients receiving both PSV23/PCV13 had reduced risks of developing secondary bacterial pneumonia, all-cause mortality, and respiratory mortality. The results were summarized in Supplementary Table S2. The findings supported the consistent benefits of PSV23/PCV13 in this patient subgroup, and, once again, supported the need for pneumococcal vaccination in these patients.

We found 3027 patients with pre-existing chronic airway diseases, and who did not receive an annual seasonal influenza vaccine in the past 12 months before the index admission, as well as PSV23 and PCV13. These patients had statistically significantly increased risks of all-cause mortality and respiratory mortality, when compared with the ‘influenza vaccine only’ subgroup. This suggested that influenza vaccines offer a mortality reduction for patients with pre-existing chronic airway diseases. The results were summarized in Supplementary Table S3.

### 3.5. Subgroup Analysis

A subgroup analysis was performed in patients who received the seasonal influenza vaccine that matched the season, and also those who received the wrong season’s vaccine; 1888 (61.6%) patients received the seasonal influenza vaccine that matched to the season and 1178 (38.4%) received the wrong season’s vaccine.

Among those who received the appropriate seasonal influenza vaccine, protection for secondary bacterial pneumonia, all-cause mortality, and respiratory mortality was observed in the ‘both PCV13/PSV23’ and ‘PCV13 only’ group. Protection was not observed in the PSV23 group.

The aOR for secondary bacterial pneumonia was 0.21 (95% CI = 0.05–0.90, *p* = 0.035) for the ‘both PCV13/PSV23’ group and 0.31 (95% CI = 0.16–0.59, *p* < 0.001) for the ‘PCV13 only’ group. The aOR for all-cause mortality was 0.22 (95% CI = 0.05–0.91, *p* = 0.037) for the ‘both PCV13/PSV23’ group and 0.32 (95% CI = 0.17–0.60, *p* < 0.001) for the ‘PCV13 only’ group. The aOR for respiratory mortality was 0.07 (95% CI = 0.00–0.48, *p* < 0.01) for the ‘both PCV13/PSV23’ group and 0.39 (95% CI = 0.19–0.74, *p* < 0.01) for ‘PCV13 only’ group.

Among those who received the wrong season’s vaccine, protection against all-cause mortality and respiratory mortality were observed in the ‘both PCV13/PSV23’ and ‘PCV13 only’ group. A protective effect on secondary bacterial pneumonia was not observed. Protection was not observed in the PSV23 group.

The aOR for all-cause mortality was 0.22 (95% CI = 0.05–0.91, *p* = 0.037) for the ‘both PCV13/PSV23’ group and 0.32 (95% CI = 0.17–0.60, *p* < 0.001) for the ‘PCV13 only’ group. The aOR for respiratory mortality was 0.01 (95% CI = 0.00–0.43, *p* = 0.01) for the ‘both PCV13/PSV23’ group and 1.13 (95% CI = 0.54–2.34, *p* = 0.75) for the ‘PCV13 only’ group.

A subgroup analysis was also performed on the patients who received PCV13 first and PSV23 first. There was no statistically significant difference in the primary and secondary outcomes between these two groups. The aOR was 1.04 (95% CI = 0.61–1.75, *p* = 0.89) for secondary bacterial pneumonia, 2.85 (0.17–482.6, *p* = 0.51) for all-cause mortality, 1.41 (95% CI = 0.40–5.00, *p* = 0.60) for secondary pneumococcal pneumonia, 0.66 (95% = 0.37–1.16, *p* = 0.15) for SRF, and 0.84 (0.02–117.50, *p* = 0.91) for all-cause ICU admission. There was no respiratory mortality and IPD in this subgroup.

## 4. Discussion

Our study suggested that, in adult patients with chronic airway diseases who had already received a seasonal influenza vaccination, those receiving both PSV23/PCV13 had the lowest risk of adverse outcomes, including secondary bacterial pneumonia, respiratory-related mortality, and all-cause mortality, when they were admitted for influenza. Receiving PSV23 was inadequate for protecting the patients from developing these adverse outcomes, while PCV13 alone can prevent all-cause mortality and respiratory-related mortality, but not the development of secondary bacterial pneumonia. The results concur with previously reported benefits from pneumococcal vaccines among patients with chronic airway diseases, reinforcing the need for adequate vaccination coverage among these high-risk patients before influenza outbreaks [10,11,21].

Patients with chronic airway diseases are well-known to be at risk of developing complications when they have influenza. And influenza can also cause the exacerbation of their underlying conditions. Secondary bacterial pneumonia was also reported to be more common in these patients when they had influenza. Hence, the importance of vaccination to protect against various infective agents cannot be over-emphasized. However, vaccine reluctance and fatigue remain a global problem, especially when more and more vaccines become available among patients with chronic diseases. In an Italian study, among prioritized at-risk groups identified by the national vaccination campaign, the uptake of pneumococcal vaccine was 33.7% among subjects aged ≥65, 48.4% among subjects with lung disease, 46.6% among subjects with cardiovascular disease, and 53.7% among subjects with DM [27]. The number in this Italian study is close to the number in our cohort. It has been reported that patients with chronic airway diseases showed hesitancy towards vaccination [28,29]. And researchers identified that subjects with more comorbidities, and more symptomatic and more frequent acute COPD exacerbations were likely to be hesitant towards vaccines [30]. The problems will be more exaggerated among these patients when more and more vaccines become available and are being recommended for them, such as severe acute respiratory syndrome coronavirus 2 (SARS-CoV-2), pertussis, respiratory syncytial virus, and herpes zoster vaccine. As such, the huge burden of vaccines does carry both a financial and psychological burden to them, when the patients are advised to follow the recommendations to receive all these vaccines, in which some of them, such as the pertussis, respiratory syncytial virus, and herpes zoster vaccine, are not covered by the Government Vaccination Programme and patients are required to pay for the vaccination costs. In light of this, clinicians should properly recommend and educate these patients to have timely and adequate vaccination, at least for those vaccines that have been well-demonstrated to have clinical benefits and are also free of charge. Among all vaccines, pneumococcal vaccines have a well-demonstrated protective effect among patients with chronic respiratory diseases. In a meta-analysis of 12 randomized trials evaluating the efficacy of PSV23 in 2171 patients with COPD, 5 trials revealed a reduction in the rate of community-acquired pneumonia with vaccination, and 4 trials showed a reduction in the rate of COPD exacerbation [31]. In our study, we focused on another aspect of chronic respiratory diseases, in which pneumococcal vaccines, in particular, receiving PCV13/PSV23, could reduce secondary bacterial pneumonia and mortality. The results from our study are consistent with prior studies showing the protective effect of pneumococcal vaccines in chronic respiratory diseases [32].

One point to note in the study is that the vaccination uptake rate is low in the cohort. While there were 6182 subjects with chronic airway diseases, less than half of them received a seasonal influenza vaccine and around half of the patients who received a seasonal influenza vaccine received any pneumococcal vaccine. And the number of subjects who received both PSV23/PCV13 was even lower. The low vaccine uptake rate is alarming. The introduction of PCV20, which can be administered as a single dose for these patients to complete their pneumococcal vaccination, may be simpler than other approaches [33,34,35].

Another important point to note is that there were significantly more patients on ICS in the PCV13/PSC23 and ‘PCV13 only’ groups. Yet, they had less secondary bacterial pneumonia than the ‘no vaccine’ and PSV23 groups. ICS is well-reported to be associated with pneumonia, especially in COPD patients [36]. Despite this, these patients who have elevated risks of pneumonia still have a lower rate of secondary bacterial pneumonia. This further reinforced the benefits of PCV13/PSC23 in pneumonia protection. And the results are still significant after the use of ICS being adjusted in the multivariate analysis.

Our study did not show protection against IPD, which is a rare event; hence, we did not have significant power to detect the incidence. Regarding secondary bacterial pneumonia, the majority was due to micro-organisms other than *S*. *pneumoniae* and we failed to demonstrate the reduction in pneumococcal pneumonia in this study. This could be related to the liberal use of empirical antibiotics in this group of patients who have chronic airway diseases upon presentation. In our cohort, two patients with secondary pneumococcal pneumonia had a positive urine pneumococcal antigen test result but a negative sputum culture for *S. pneumoniae*. There were 94 subjects who developed secondary bacterial pneumonia based on the clinical criteria with no identifiable bacterial pathogen from the respiratory specimens. Other micro-organisms identified, such as *Stenotrophomonas maltophilia* (*S. maltophilia*) in 169 of the patients, could also emerge after the exposure to antibiotics, instead of the original culprit for secondary bacterial pneumonia. The early liberal use of antibiotics could reduce the detection rate of *S*. *pneumoniae* in the respiratory specimens of the patients after antibiotics exposure [37]. This could explain the overall secondary bacterial pneumonia risk but not a pneumococcal pneumonia risk, as some of the non-pneumococcal secondary bacterial pneumonia could actually represent pneumococcal pneumonia that failed to grow *S. pneumoniae* in the respiratory specimens from a prior antibiotic exposure. A prior study also suggested that the diagnosis of pneumococcal disease may rely on the urine pneumococcal antigen test [38]. A study conducted with IPD reported that *S. pneumoniae* was isolated from at least one bacteriological sample in 48.6% of patients, but, in 51.4%, the diagnosis was only based on the results of the pneumococcal antigen urinary test [38]. The use of a urine pneumococcal antigen test is needed if the clinical suspicion is high, especially if the patients had already received antibiotics which may not allow the recovery of *S. pneumoniae* in respiratory tract specimens. Another phenomenon we observed, which is also consistent with a previous report [8], is that, in this cohort with predominantly COPD patients, Gram-negative organisms were common pathogens causing secondary bacterial pneumonia. *Klebsiella pneumoniae*, *P. aeruginosa*, *Acinetobacter bumannii,* and *S. maltophilia* are the common Gram-negative organisms identified in our cohort.

Another interesting phenomenon we observed is that pneumococcal vaccines might prevent secondary bacterial pneumonia that were not due to *S. pneumoniae*. The all-cause pneumonia protection effect from pneumococcal vaccines had been reported in large-scale population-based studies [39,40,41,42,43,44]. One of the explanations of the observation is that *S. pneumoniae* could not be recovered after the exposure to antibiotics. Another possible mechanism can be related to trained immunity, which may also explain how the cross-protection effects from the Bacille Calmette–Guérin (BCG) vaccination on the COVID-19 SARS-CoV-2 vaccination could also trigger trained immunity and offer protection against tuberculosis [45,46]. The development of the T-cell and cellular immune response after pneumococcal vaccination might trigger long-term memory, offering the potential cross-pathogen protection. Yet, this phenomenon has to be properly studied.

Our cohort has a relatively low ICU admission rate compared to other reports [47,48,49]. In a Spanish study, the ICU admission rate was 7.4%, with the highest rate in those aged 40–59 years (13.9%), with a large gap observed between that age group and the oldest patients (2.5%) [47]. The differences can firstly be explained by the differences in the study population, in which our cohort mostly comprise older patients with a mean age of 76.2 ± 10.8 years who have different co-morbidities. They are considered to have a low priority of ICU care despite the complications developed from influenza. The patients who had severe diseases such as respiratory failure were also managed in the designated ventilator unit and isolation ward in Hong Kong instead of the ICU as the number of beds in the ICU are limited. The differences in practice could also explain the differences between the rate of ICU admission with the previous literature.

Our study did have some limitations to address. Firstly, missing data were present in the ethnic groups which were managed by multiple imputations. Secondly, regarding the outcome on ICU admission, there was a potential selection bias as older patients with more medical co-morbidities might not be admitted to the ICU despite having a more severe disease. In this cohort, there was a significant proportion of patients who developed SRF but were not admitted to the ICU. This could be related to the selection bias based on the patients’ underlying health status, which resulted in them being rejected by the ICU despite the NIV/IMV for SRF. Thirdly, the Sequential Organ Failure Assessment (SOFA) Score, which reflects the disease severity, was not available. The clinical outcomes were chosen to be the outcomes of interest instead. In this cohort, the overall incidence of IPD is low. The lack of statistical difference in IPD among patients on different pneumococcal vaccines could be related to the lack of power to detect the difference. In our study, only 411 patients had a urine pneumococcal antigen test performed. The low usage of the urine pneumococcal antigen test is related to the costs of the test, and it is not readily available in all centres. Some centres will need the endorsement of microbiologists or infectious disease specialists before the test can be performed. The low usage rate of the urine pneumococcal antigen test might underestimate the burden of secondary pneumococcal pneumonia in our study, which was based on a sputum culture alone. Including the urine pneumococcal antigen test as a standard practice should be considered in order to allow for precision medicine and could also allow the appropriate step-down of broad-spectrum antibiotics [50].

## 5. Conclusions

Completed pneumococcal vaccination with PSV23/PCV13 confer protection against secondary bacterial pneumonia, all-cause mortality, and respiratory cause of mortality among patients with chronic airway diseases with a prior seasonal influenza vaccine upon hospitalization for influenza.

## Figures and Tables

**Table 1:** Baseline demographic and clinical characteristics for all the patients included in this study.

		Whole Cohort	No PSV23/PCV13	PSV23 Only	PCV13 Only	Both PSV23/PCV13	*p*-Value #
Number of subjects (n)		3066	1521	133	1091	321	-
Age, years (Mean ± SD)		76.2 ± 10.8	75.7 ± 12.4	75.1 ± 8.9	78.0 ± 8.4	72.4 ± 9.7	<0.001 *
Male (n, (%))		2152 (70.2%)	1008 (66.3%)	103 (77.4%)	791 (72.5%)	250 (77.9%)	<0.001 *
Ethnic group (n, (%))	Chinese	3041 (99.2%)	1504 (98.9%)	132 (99.2%)	1086 (99.5%)	319 (99.4%)	0.290
	Northeast Asian	1 (0.0%)	1 (0.1%)	0 (0%)	0 (0%)	0 (0%)	
	Southeast Asian	6 (0.2%)	3 (0.2%)	1 (0.8%)	2 (0.2%)	0 (0%)	
	South Asian	12 (0.4%)	10 (0.7%)	0 (0%)	2 (0.2%)	0 (0%)	
	Caucasian	2 (0.1%)	1 (0.1%)	0 (0.0%)	1 (0.1%)	0 (0.0%)	
	Others	4 (0.1%)	2 (0.1%)	0 (0%)	0 (0%)	2 (0.6%)	
eGFR, mL/min/1.73 m^2^ (Mean ± SD)		63.5 ± 23.6	63.8 ± 24.0	63.1 ± 20.0	62.7 ± 23.5	64.7 ± 23.8	0.51
Underlying chronic airway disease (n, (%))	Asthma	1037 (33.8%)	534 (20.1%)	40 (30.1%)	366 (33.5%)	97 (30.2%)	0.277
	Bronchiectasis	517 (16.9%)	271 (17.8%)	20 (15.0%)	173 (15.9%)	53 (16.5%)	0.548
	COPD	2123 (69.2%)	1002 (65.9%)	96 (72.2%)	787 (72.1%)	238 (74.1%)	<0.001 *
Cardiovascular diseases (n, (%))		1340 (43.7%)	702 (46.2%)	53 (39.8%)	477 (43.7%)	108 (33.6%)	<0.0018
Diabetes mellitus (n (%))		781 (25.5%)	394 (25.9%)	31 (23.3%)	295 (27.0%)	61 (19.0%)	0.030 *
History of malignancies (n, (%))		266 (8.7%)	150 (9.9%)	16 (12.0%)	83 (7.6%)	17 (5.3%)	0.013 *
CCI (Mean ± SD)		4.7 ± 2.0	4.8 ± 2.2	4.6 ± 1.9	4.9 ± 1.9	4.0 ± 1.9	<0.001 *
LAMAs (n, (%))		1272 (57.8%)	841 (55.3%)	80 (60.2%)	656 (60.1%)	195 (60.7%)	0.051
LABAs (n, (%))		2102 (68.6%)	1040 (68.4%)	93 (69.9%)	751 (68.8%)	218 (67.9%)	0.971
ICS (n, (%))		2131 (69.5%)	962 (63.2%)	89 (66.9%)	837 (76.7%)	243 (75.7%)	<0.001 *

#: Comparing the four subgroups, with the ‘No PSV23/PCV13’ being the group of reference. SD = standard deviation; * = statistically significant, eGFR = estimated glomerular filtration rate, CCI = Charlson comorbidity index, LABAs = long-acting beta-agonists, LAMAs = Long-acting muscarinic antagonists, ICS = Inhaled corticosteroid.

## Data Availability

All available data are presented in the manuscript and no additional data will be provided.

## Supplementary Materials

The following supporting information can be downloaded at: https://www.mdpi.com/article/10.3390/vaccines12070704/s1, Supplementary Table S1: Microbiology of the micro-organism isolated in secondary bacterial pneumonia; Supplementary Table S2: Risks of developing severe in-hospital outcomes in subgroup age ≥ 65; Supplementary Table S3: Risks of developing severe in-hospital outcomes among patients from different subgroups, including patients who did not receive influenza and pneumococcal vaccines; Supplementary Figure S1: Annual hospitalization number of the patients with pre-existing chronic airway diseases and received annual seasonal influenza vaccine in the past 12 months before the index admission.
