# Supplementary material for: Protective Effects from Prior Pneumococcal Vaccination in Patients with Chronic Airway Diseases during Hospitalization for Influenza—A Territory-Wide Study
Source: Vaccines (Basel). 2024 Jun 23;12(7):704. doi: 10.3390/vaccines12070704 (PMC11281690; doi:10.3390/vaccines12070704)
Supplement: Supplementary file 1 [file vaccines-12-00704-s001.zip › vaccines-3026835-supplementary.pdf]

Supplementary Table S1. Microbiology of the microorganism isolated in secondary bacterial pneumonia.

**Microorganism**	**Number (%)**
Streptococcus pneumoniae	148 (8.1%)
Other Streptococcus species	2 (0.1%)
Staphylococcus aureus	332 (18.3%)
Haemophillus influenzae	138 (7.6%)
Moraxella catarrhalis	196 (10.8%)
Klebsiella pneumoniae	52 (2.9%)
Pseudomonas aeruginosa	174 (9.6%)
Stenotrophomonas maltophilia	169 (9.3%)
Acinetobacter bumannii	27 (1.5%)

Legionella pneumophila	2 (0.1%)
Other microorganisms	484 (26.6%)
Commensals/no growth	94 (5.2%)

Supplementary Table S2. Risks of developing severe in-hospital outcomes in subgroup age ≥ 65.

	No PSV23/PCV13 (n = 1261)	PSV23 only (n = 124)		PCV13 only (n = 1017)		Both PSV23/PCV13 (n = 262)	
	Number of subjects (%)	Number of subjects (%)	aOR (95% CI) p- value	Number of subjects (%)	aOR (95% CI) p- value	Number of subjects (%)	aOR (95% CI) p- value
**Secondary bacterial pneumonia**	805 (63.8%)	83 (66.9%)	1.31<br>0.88 – 1.97<br>0.19	607 (59.7%)	0.89<br>0.75 – 1.07<br>0.21	132 (50.4%)	0.71<br>0.54 – 0.95<br>0.019*
**All-cause mortality**	84 (6.7%)	8 (6.5%)	1.16<br>0.54 – 2.52<br>0.70	29 (2.9%)	0.52<br>0.33 – 0.81<br>0.004*	2 (0.8%)	0.11<br>0.02 - 0.53<br>0.006*
**Respiratory cause of mortality**	64 (5.1%)	6 (4.8%)	1.18<br>0.46 - 2.58<br>0.71	26 (2.6%)	0.60<br>0.37 - 0.96<br>0.03*	0 (0%)	0.03<br>0.00 - 0.26<br><0.001*

**Pneumococcal pneumonia**	53 (4.2%)	6 (4.8%)	0.98<br>0.41 – 2.35<br>0.96	56 (5.5%)	1.22<br>0.82 – 1.82	13 (5.0%)	0.89<br>0.46 – 1.72<br>0.73
**IPD**	3 (0.2%)	0 (0%)	1.05<br>0.01 - 11.29<br>0.97	1 (0.1%)	0.54<br>0.05 - 3.40<br>0.52	0 (0%)	0.67<br>0.00 - 7.69<br>0.78
**SRF**	421 (33.4%)	46 (37.1%)	1.22<br>0.81 – 1.82	331 (32.5%)	0.98<br>0.81 – 1.19<br>0.84	88 (33.6%)	1.12<br>0.83 – 1.52<br>0.47
**ICU admission**	7 (0.6%)	1 (0.8%)	1.93<br>0.20 - 9.35<br>0.50	5 (0.5%)	1.03<br>0.31 - 3.23<br>0.97	0 (0%)	0.38<br>0.00 - 3.11<br>0.44

*: Statistically significant; IPD: invasive pneumococcal disease; SRF: Severe respiratory failure requiring invasive or non-invasive mechanical ventilation; ICU: Intensive care unit

Adjusted for age; sex; ethnicity; baseline Charlson comorbidity index; which chronic airway diseases the patient is suffering from; presence of DM, cardio-/cerebro-vascular diseases, history of malignancies and the use of long-acting beta-agonists, long-acting muscarinic antagonists and inhaled corticosteroid

Supplementary Table S3. Risks of developing severe in-hospital outcomes among patients from different subgroups, including patients who did not receive influenza and pneumococcal vaccines.

	**Influenza vaccine but no PSV23/PCV13 (n = 1521)**	**No influenza/ PSV23/PCV13 (n = 3027)**		**PSV23 only (n = 133)**		**PCV13 only (n = 1091)**		**Both PSV23/PCV13 (n = 321)**	
	Number of subjects (%)	Number of subjects (%)	aOR (95% CI) p- value	Number of subjects (%)	aOR (95% CI) p- value	Number of subjects (%)	aOR (95% CI) p- value	Number of subjects (%)	aOR (95% CI) p- value
**Secondary bacterial pneumonia**	928 (61.0%)	1798 (59.4%)	1.16 (1.01 – 1.34) 0.036	86 (64.7%)	1.16 (0.79 – 1.71) 0.35	640 (58.7%)	0.86 (0.73 – 1.02) 0.13	164 (51.1%)	0.74 (0.57 – 0.95), 0.019
**All-cause mortality**	87 (5.7%)	266 (8.8%)	1.52 (1.17 – 1.97) 0.006	8 (6.0%)	1.19 (0.55 – 2.56) 0.73	30 (2.7%)	0.53 (0.34 – 0.83) 0.005	2 (0.6%)	0.12 (0.03 – 0.53) 0.005
**Respiratory cause of mortality**	67 (4.4%)	199 (6.6%)	1.39 (1.04 – 1.87) 0.028	6 (4.5%)	1.18 (0.46 – 2.56) 0.84	27 (2.5%)	0.61 (0.38 – 0.97) 0.036	0 (0%)	0.04 (0.00 – 0.53,) 0.0038)
**Pneumococcal pneumonia**	66 (4.3%)	87 (2.9%)	0.73 (0.52 – 1.02) 0.06	6 (4.5%)	0.92 (0.34 – 2.18) 0.85	58 (5.3%)	1.16 (0.80 – 1.69) 0.42	18 (5.6%)	1.15 (0.67 – 1.98) 0.62
**IPD**	3 (0.2%)	4 (0.1%)	0.58 (0.13 - 2.71) 0.47	0 (0%)	1.48 (0.01 - 15.39) 0.80	1 (0.1%)	0.66 (0.06 - 4.12) 0.66	0 (0%)	0.65 (0.00 - 6.92) 0.77

**SRF**	509 (33.5%)	308 (30.0%)	1.00 (0.84 – 1.44) 0.99	47 (35.3%)	1.08 (0.73 – 1.60) 0.69	359 (32.9%)	0.99 (0.83 – 1.17) 0.87	109 (34.0%)	1.10 (0.84 - 1.44) 0.48
**ICU admission**	10 (0.7%)	23 (0.8%)	1.10 (0.51 – 2.39) 0.80	1 (0.8%)	1.17 (0.15 – 9.28) 0.88	7 (0.6%)	1.24 (0.46 – 3.32) 0.67	1 (0.3%)	0.50 (0.06 – 3.98) 0.51

IPD: invasive pneumococcal disease; SRF: Severe respiratory failure requiring invasive or non-invasive mechanical ventilation; ICU: Intensive care unit

Adjusted for age; sex; ethnicity; baseline Charlson comorbidity index; which chronic airway diseases the patient is suffering from; presence of DM, cardio-/cerebro-vascular diseases, history of malignancy and the use of long-acting beta-agonists, long-acting muscarinic antagonists and inhaled corticosteroid

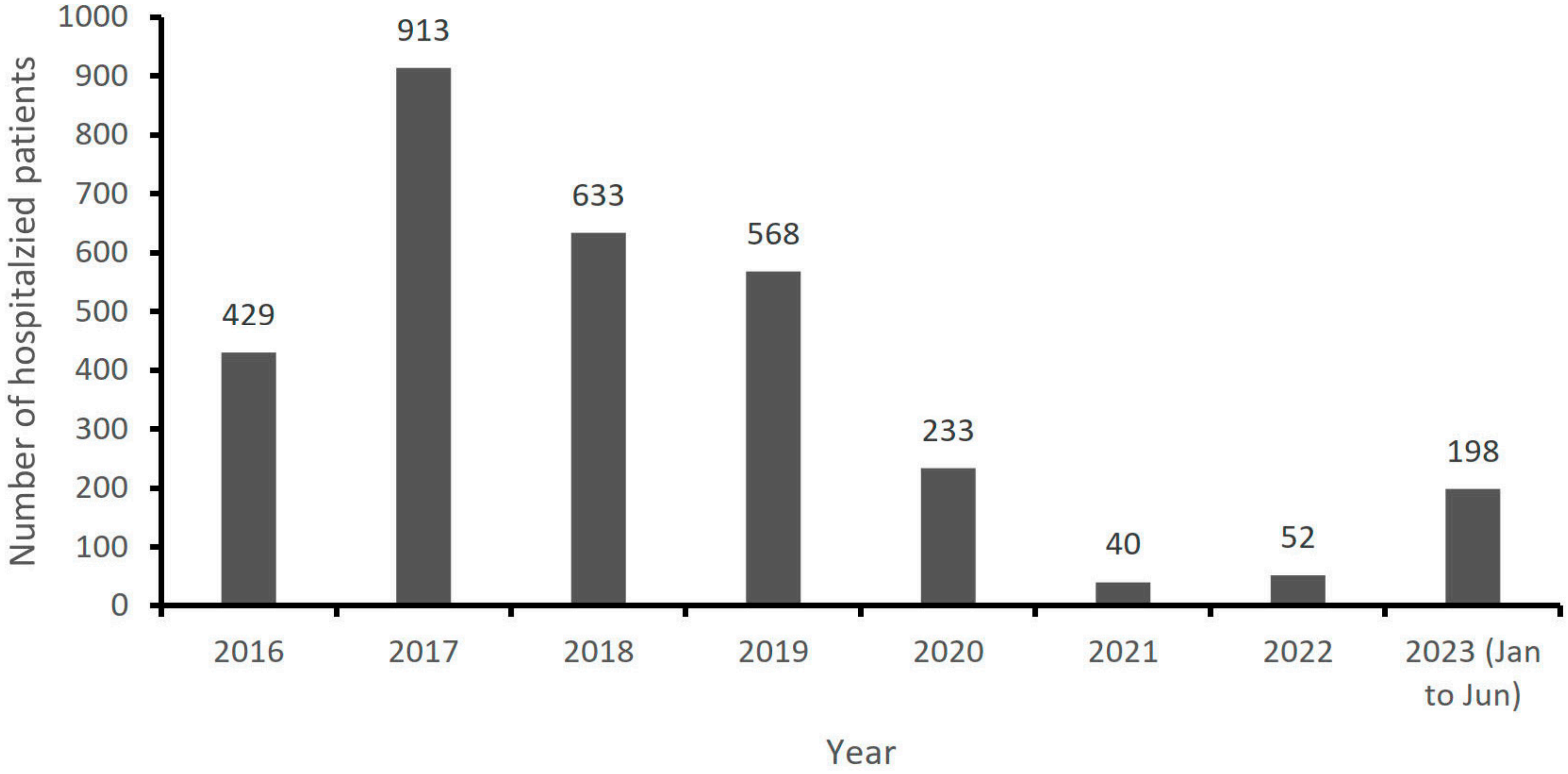

Supplementary Figure S1. Annual hospitalization number of the patients with pre-existing chronic airway diseases and received annual seasonal influenza vaccine in the past 12 months before the index admission.
